# Disturbance in the potential cardiovascular–bone–skeletal muscle axis and morbidity and mortality in patients undergoing haemodialysis: the Q-Cohort Study

**DOI:** 10.1093/ckj/sfae154

**Published:** 2024-06-17

**Authors:** Hokuto Arase, Shunsuke Yamada, Masatomo Taniguchi, Hiroaki Ooboshi, Kazuhiko Tsuruya, Takanari Kitazono, Toshiaki Nakano

**Affiliations:** Department of Medicine and Clinical Science, Graduate School of Medical Sciences, Kyushu University, Higashi-Ku, Fukuoka, Japan; Department of Nephrology, NHO Fukuokahigashi Medical Center, Koga, Japan; Department of Medicine and Clinical Science, Graduate School of Medical Sciences, Kyushu University, Higashi-Ku, Fukuoka, Japan; Fukuoka Renal Clinic, Chuo-Ku, Fukuoka, Japan; Department of Internal Medicine, Fukuoka Dental College, Sawara-Ku, Fukuoka, Japan; Department of Nephrology, Nara Medical University, Kashihara, Nara, Japan; Department of Medicine and Clinical Science, Graduate School of Medical Sciences, Kyushu University, Higashi-Ku, Fukuoka, Japan; Department of Medicine and Clinical Science, Graduate School of Medical Sciences, Kyushu University, Higashi-Ku, Fukuoka, Japan

**Keywords:** bone fracture, cardiovascular, haemodialysis, sarcopenia, skeletal muscle

## Abstract

**Background:**

Disturbances in the cardiovascular system, bone and skeletal muscle are independent risk factors for death among patients receiving haemodialysis (HD). However, the combined impact of disorders of these three organs on morbidity and mortality is unclear in the HD population.

**Methods:**

A total of 3031 Japanese patients on maintenance HD were prospectively followed. The outcomes were all-cause mortality, major adverse cardiovascular events (MACE) and bone fracture. Patients were divided into four groups (G1–G4) according to the baseline number of diseased organs represented as histories of cardiovascular disease and bone fractures and the presence of low skeletal muscle mass as follows: G1, no organ; G2, one organ; G3, two organs; G4, three organs. Multivariable-adjusted survival models were used to analyse associations between the number of diseased organs and outcomes.

**Results:**

During a 4-year follow-up, 499 deaths, 540 MACE and 140 bone fractures occurred. In the Cox proportional hazards model, the risk for all-cause mortality was significantly higher in G2, G3 and G4 than in G1 as the reference {hazard ratio: G2, 2.16 [95% confidence interval (CI) 1.65–2.84], G3, 3.10 [95% CI 2.27–4.23] and G4, 3.11 [95% CI 1.89–5.14]}. Similarly, the risks for developing MACE and bone fractures were significantly elevated as the number of organ disorders increased.

**Conclusions:**

Multiple disorders of the cardiovascular–bone–skeletal muscle axis are strong predictors of morbidity and mortality in patients undergoing HD.

## Graphical Abstract

**Graphical Abstract:**
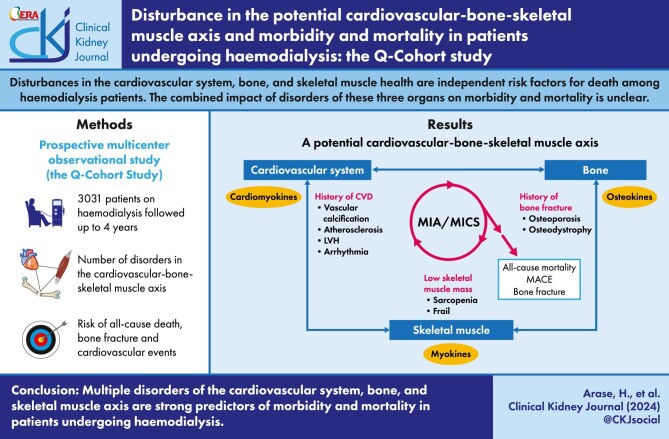

KEY LEARNING POINTS **What was known:**

- The cardiovascular system, bone and skeletal muscle work interdependently by the integrated actions of humoral factors and mechanical forces for each organ's coordinated development and maintenance.
- Diseases of the cardiovascular system, bone and skeletal muscle have become more common in the aging haemodialysis (HD) population and each organ disorder independently causes a decline in the activity of daily living and quality of life but elevates the risk for mortality.
- The bone–vascular axis or calcification paradox, which describes a significant association between derangements in the cardiovascular system and bone, is now a widely accepted concept, especially in the field of chronic kidney disease.

**This study adds:**

- We propose that potential crosstalk between diseased organs has a pivotal role in the vicious cycle of derangement of the cardiovascular system, bone and skeletal muscle in the HD population.
- The present study investigated whether the cardiovascular–bone–skeletal muscle axis, an extension of the concept of the bone–vascular axis should cover the musculoskeletal system, exists in patients on maintenance HD and assessed the overall impact of this axis on clinically relevant outcomes.

**Potential impact:**

- Combined disturbances in cardiovascular, bone and skeletal muscle health are significantly associated with higher risks for morbidity and mortality in patients on maintenance HD.
- Integrated intervention in this cardiovascular–bone–skeletal muscle axis by pharmacological or non-pharmacological treatments may greatly improve outcomes in the HD population.

## INTRODUCTION

Bone and skeletal muscle are the two primary components of the normal musculoskeletal system that confer the basis for form, support, stability and movement of the body. These two organs work interdependently and provide an integrated foundation for the musculoskeletal system. Their interactions are mainly mediated by the integrated actions of humoral factors and mechanical forces [1–3]. Obviously, an adequate supply of oxygen, nutrients and endocrine factors with sufficient blood flow from the cardiovascular system is also vital for maintaining normal metabolism in these organs [4, 5]. Conversely, cytokines secreted by bone and skeletal muscle support the normal function and structure of the cardiovascular system [2, 6]. Thus there is an essential interplay between the cardiovascular system, bone and skeletal muscle for each organ's coordinated development and maintenance. Moreover, disorders of one of these organs may compromise the physiological function and structure of the other organs, leading to multiorgan dysfunction.

Diseases of the cardiovascular system, bone and skeletal muscle have become more common in the aging haemodialysis (HD) population [7]. Organ disorders such as ischaemic heart disease/stroke, bone fracture and sarcopenia cause a decline in the activity of daily living and quality of life while elevating the risk for mortality [8–11]. Given the tight interdependence of the cardiovascular, bone and skeletal muscle systems, it is reasonable to speculate that individuals with multiple disorders of these organs may be at a greater risk of morbidity and mortality through a vicious cycle of affected organs. One example of this is the bone–vascular axis or calcification paradox, which describes a significant association between derangements in the cardiovascular system and bone [12–14]. The coexistence of cardiovascular calcification and osteoporosis in the same patient is often referred to as the bone–vascular axis [15, 16]. A strong link between skeletal muscle and bone or the cardiovascular system has also been documented. Low skeletal muscle mass, part of the sarcopenia phenotype, was shown to elevate the risk of developing heart disease and bone fractures in haemodialyzed patients [17, 18]. On the basis of these findings, we speculated that potential crosstalk between diseased organs has a pivotal role in the vicious cycle of derangement of the cardiovascular–bone–skeletal muscle axis that affects the outcomes of HD patients. However, few studies have focused on the combined impact of disorders in these vital organs on the morbidity and mortality of the HD population.

The present study investigated whether the cardiovascular–bone–skeletal muscle axis exists, an extension of the concept of the bone–vascular axis should cover the musculoskeletal system, in patients on maintenance HD and assessed the impact of this axis on clinically relevant outcomes. To achieve these aims, we examined the association between the baseline number of disorders in the cardiovascular, bone and skeletal muscle systems with future outcomes, including mortality, major adverse cardiovascular events (MACE) and bone fractures, using the dataset of the Q-Cohort Study [17–22].

## MATERIALS AND METHODS

### Design of the Q-Cohort Study

The Q-Cohort Study was conducted as a multicentre (39 dialysis facilities), prospective, observational study of patients undergoing prevalent HD in Japan [17–22]. A total of 3598 outpatients ≥18 years of age and receiving regular HD therapy between December 2006 and December 2007 were registered and followed up until December 2010. Patients not fully followed up until the study end were also included in the analyses as ‘censored’ on the day of the final visit. In this study, 567 patients were excluded from the primary analyses because they lacked data related to their baseline characteristics and the remaining 3031 patients were analysed. This study followed the Ethics of Clinical Research (1975 Declaration of Helsinki) and was approved by the Kyushu University Hospital Institutional Review Board for Clinical Research (20-31). The study was registered in a clinical trial registry (University Hospital Medical Information Network, UMIN000000556). Written informed consent was provided by all patients before participation in the study.

### Demographics and biochemical measurements

Baseline data, including demographics and clinical characteristics at baseline, were recorded at enrolment. A detailed description of biochemical measurements was previously reported [17–22] and included in the supplementary material. The modified creatinine index, which reflects skeletal muscle mass in patients receiving maintenance HD, was calculated as follows: modified creatinine index (mg/kg/day) = 16.21 + 1.12 × [1 if male; 0 if female] − 0.06 × age (years) − 0.08 × single-pool Kt/V for urea + 0.009 × pre-dialysis serum creatinine (μmol/l) [17, 18, 21, 23].

### Definition of outcomes and covariates

**Figure 1:**
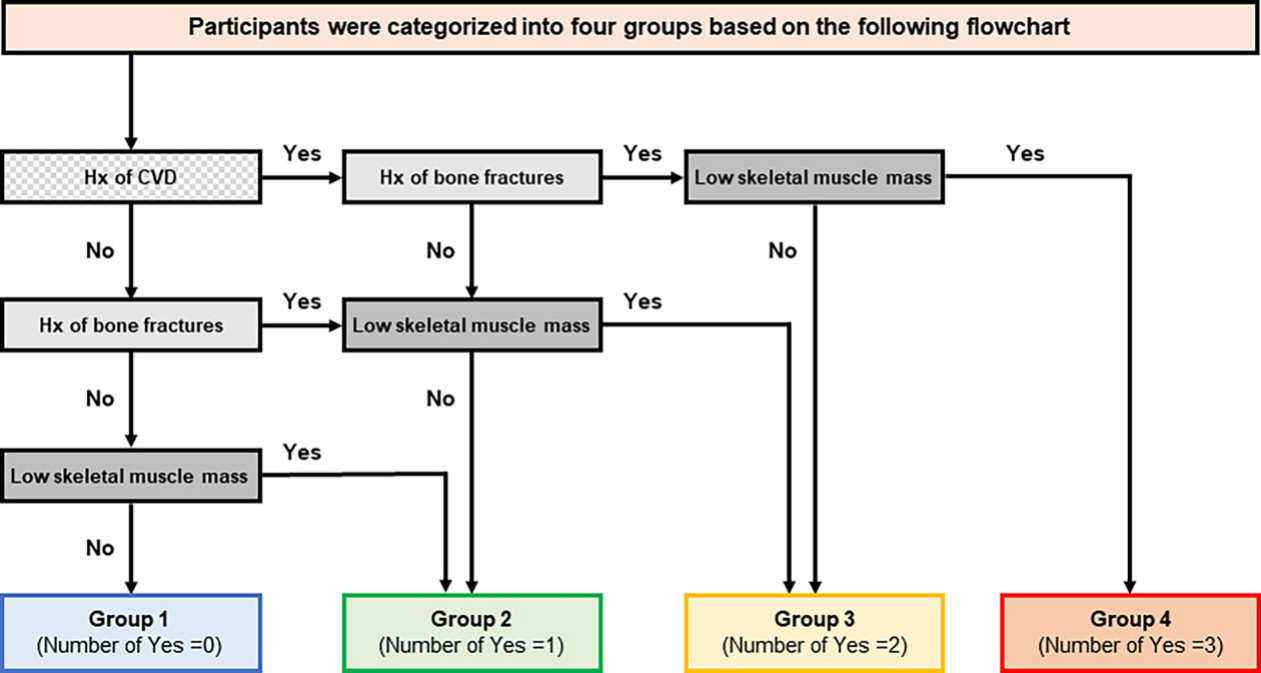
A flow chart of the categorization of the participants. Patients were divided into four groups according to the baseline number of diseased organs represented as a history of cardiovascular disease, a history of bone fractures and the presence of low skeletal muscle mass. Patients who had no diseased organ were categorized into group 1, those who had one diseased organ were categorized into group 2, those who had two diseased organs were categorized into group 3 and those who had three diseased organs were categorized into group 4. CVDs: cardiovascular diseases; Hx: history.

The primary outcome was all-cause mortality and the secondary outcomes were the development of MACE and bone fracture. In this study, MACE was defined as combined events, including the first-ever development of myocardial infarction, hospitalization for unstable angina, coronary intervention (coronary artery bypass surgery or angioplasty), hospitalization for heart failure and the development of stroke composed of brain infarction and haemorrhage [22]. A bone fracture was defined as clinical symptoms and fracture of bone confirmed by imaging at any site [18]. Previous studies have described the definitions of each disease in detail [18, 19, 22]. The covariate of interest was the prevalence of the histories of cardiovascular disease and bone fractures and the presence of low skeletal muscle mass at enrolment, all of which were considered to be disorders of the cardiovascular system, bone and skeletal muscle, respectively, in this study. Low skeletal muscle mass was defined in patients with a low modified creatinine index below the thresholds for men and women at baseline, based on previous research demonstrating the discriminative ability of the modified creatinine index for sarcopenia in a maintenance HD population [24]. Briefly, Kakita *et al.* [24] diagnosed haemodialyzed patients as having sarcopenia based on the low skeletal muscle mass evaluated by bioimpedance analysis and low muscle strength or low physical performance according to the Asian Working Group for Sarcopenia 2019 criteria [25]. The modified creatinine index had equal or superior diagnostic performance for sarcopenia compared with other common assessment methods, including SARC-F (strength, assistance with walking, rising from a chair, climbing stairs and falls) or SARC-CalF (SARC-F with calf circumference) score [24]. The cut-off values of the modified creatinine index used to identify patients with sarcopenia were 21.3 mg/kg/day for men and 18.8 mg/kg/day for women. When these cut-off values were applied to patients in our study, the prevalence of low skeletal muscle mass in this study was similar to the prevalence of sarcopenia in previous studies [24, 26] (Supplementary Figure S1). Patients were divided into four groups (G1–G4) according to the baseline number of diseased organs represented as histories of cardiovascular disease and bone fractures and the presence of low skeletal muscle mass as follows: G1, no diseased organ; G2, one diseased organ; G3, two diseased organs; and G4, three diseased organs (Fig. 1).

### Statistical analysis

For the baseline characteristics, continuous variables are described as the median and interquartile range (IQR) and categorical data are expressed as numbers and percentages. The association between the prevalence of histories of cardiovascular disease and bone fractures and the presence of low skeletal muscle mass was analysed using the χ^2^ test. To compare the distribution of baseline characteristics in each group, trend analyses were performed using the Jonckheere–Terpstra test for continuous variables and the Cochran–Armitage test for categorical variables. The Kaplan–Meier method with logrank test was used to examine the event-free survival rates for all-cause death, MACE or bone fracture in each group. The Cox proportional hazards model was used to investigate sex- and age-adjusted and multivariable-adjusted hazard ratios (HRs) and 95% confidence intervals (CIs) for outcomes in each group. The Fine–Gray proportional subdistribution hazards model setting all-cause death as a competing risk was also performed to assess multivariable-adjusted HRs for MACE and bone fracture (the competing risk model). The assumption of proportional hazards was checked graphically using log cumulative hazard plots for each outcome in each group. The following covariates were used in the multivariable-adjusted models for all-cause mortality and the development of MACE: age; sex; presence of diabetic nephropathy; dialysis vintage; dialysis time per session; systolic blood pressure; cardiothoracic ratio; normalized protein catabolic rate (nPCR); Kt/V for urea; body mass index (BMI); blood haemoglobin; serum albumin, total cholesterol, corrected calcium, phosphate and alkaline phosphatase; log serum C-reactive protein (CRP); log serum intact parathyroid hormone (iPTH) and use of anti-hypertensives, phosphate binders and vitamin D receptor activators (VDRAs). The following covariates were used in the multivariable-adjusted models for the development of bone fracture: age; sex; presence of diabetic nephropathy; dialysis vintage; BMI; serum albumin, corrected calcium, phosphate and alkaline phosphatase; log serum CRP; log serum iPTH and use of phosphate binders and VDRAs. Variables in the multivariable-adjusted models were selected based on *a priori* clinical judgment and previous reports. Because the incidence of newly developed bone fractures during the observation period was relatively small compared with other outcomes, fewer covariates were selected in the multivariable analyses. A two-tailed *P-*value <.05 was considered statistically significant in all analyses. Statistical analyses were performed using JMP version 14.2.0 (SAS Institute, Cary, NC, USA) and R software version 3.0.2 (http://cran.rproject.org).

## RESULTS

### Association among cardiovascular, bone and skeletal muscle disorders

**Figure 2:**
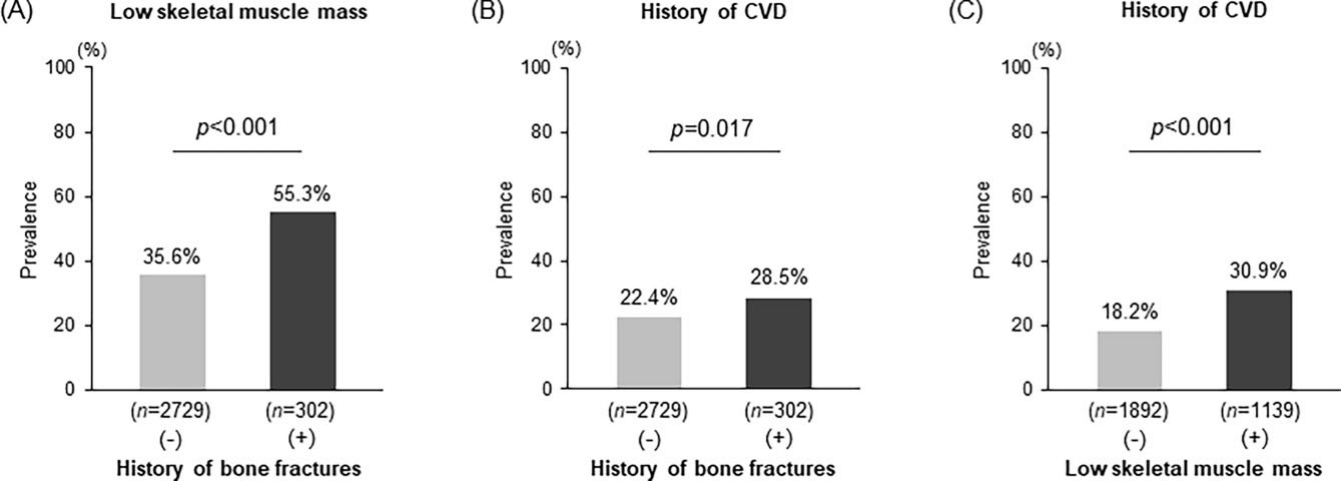
Associations between **(A)** a history of bone fractures and the presence of low skeletal muscle mass, **(B)** histories of bone fractures and cardiovascular disease and **(C)** the presence of low skeletal muscle mass and a history of cardiovascular disease. The χ^2^ test was used to analyse statistical differences. A two-tailed *P*-value <.05 was considered statistically significant.

First, the association among the prevalence of histories of cardiovascular disease and bone fractures and the presence of low skeletal muscle mass was analysed. The prevalence of a history of bone fractures was significantly associated with increased rates of the simultaneous presence of low skeletal muscle mass and a history of cardiovascular disease, respectively (low skeletal muscle mass, *P* < .001; history of cardiovascular disease, *P* *= *.017; Fig. 2A and B). Furthermore, the prevalence of low skeletal muscle mass was significantly associated with an increased rate of a history of cardiovascular disease (*P* < .001; Fig. 2C).

### Classification of patients according to the number of cardiovascular, bone and skeletal muscle disorders

Patients were divided into four groups according to the following three complications: history of cardiovascular disease, history of bone fractures and the presence of low skeletal muscle mass (Fig. 1). The breakdown of each group is shown in Table 1. Cardiovascular disease and low skeletal muscle mass were more common than bone fracture in groups 2 and 3.

**Table 1:** Breakdown of each group classified according to the total number of cardiovascular, bone, and skeletal muscle disorders (*N* = 3031).

Organ disorders	Overall (*N* = 3031)	Group 1 ( *n* = 1440)	Group 2 ( *n* = 1102)	Group 3 (*n* = 431)	Group 4 (*n* = 58)
History of cardiovascular disease, *n* (%)	697 (23.0)	0 (0)	317 (28.8)	322 (74.7)	58 (100)
History of bone fractures, *n* (%)	302 (10.0)	0 (0)	107 (9.7)	137 (31.8)	58 (100)
Presence of low skeletal muscle mass, *n* (%)	1139 (37.5)	0 (0)	678 (61.5)	403 (93.5)	58 (100)

Patients with low skeletal muscle mass were defined as those with a low modified creatinine index below the threshold at baseline. In this study, the cut-off values of the modified creatinine index were 21.3 mg/kg/day for men and 18.8 mg/kg/day for women.

### Baseline characteristics of each group

The baseline characteristics of each group are shown in Table 2. Patients in group 4 were significantly older and had a higher prevalence of diabetic nephropathy, shorter dialysis vintage, shorter dialysis time per session, lower BMI and nPCR and higher cardiothoracic ratio compared with patients in group 1. The modified creatinine index was significantly lower in patients in group 4 compared with patients in group 1, regardless of sex. Blood haemoglobin and serum albumin, urea nitrogen, creatinine, corrected calcium, phosphate and iPTH were significantly lower in group 4, whereas serum CRP and alkaline phosphatase were significantly higher in group 4 compared with group 1. Patients in group 4 used phosphate binders and VDRAs significantly less frequently, whereas they used antihypertensives significantly more frequently compared with patients in group 1.

**Table 2:** Clinical backgrounds of each group at baseline (*N* = 3031).

Baseline characteristics	Group 1 ( *n* = 1440)	Group 2 ( *n* = 1102)	Group 3 ( *n* = 431)	Group 4 ( *n* = 58)	*P* for trend
Demographics and dialysis-related information					
Age (years)	58.4 (50.7–65.1)	68.2(59.5–75.9)	74.2 (68.3–75.9)	78.5 (72.0–82.8)	<.001
Female, *n* (%)	609 (42.3)	419 (38.0)	175 (40.6)	32 (55.2)	.771
Diabetic nephropathy, *n* (%)	298 (20.7)	361(32.8)	186 (43.2)	28 (48.3)	<.001
Dialysis vintage (years)	6.8 (3.0–12.9)	4.4 (1.4–10.9)	4.0 (1.6–9.0)	4.6 (1.3–9.6)	<.001
Dialysis time per session (≥5 h), *n* (%)	935 (64.9)	666 (60.4)	250 (58.0)	28 (48.3)	<.001
Kt/V for urea	1.6 (1.4–1.8)	1.6 (1.4–1.7)	1.6 (1.5–1.7)	1.6 (1.5–1.6)	.242
BMI (kg/m^2^)	21.2 (19.2–23.4)	20.8 (18.6–23.0)	19.9 (18.1–22.0)	19.8 (17.7–21.6)	<.001
nPCR (g/kg/day)	1.0 (0.9–1.1)	0.9 (0.8–1.0)	0.9 (0.8–1.0)	0.9 (0.8–1.0)	<.001
Systolic blood pressure (mmHg)	152 (138–168)	154 (140–170)	153 (136–167)	150 (140–172)	.502
Cardiothoracic ratio (%)	49.1 (46.1–52.1)	50.9 (47.7–54.5)	53.1 (48.8–56.8)	53.1 (50.1–56.7)	<.001
Modified creatinine index (mg/kg/day)					
in males (*n* = 1796)	23.6 (22.4–25.1)	20.9 (19.7–22.7)	19.8 (19.0–20.7)	19.1 (17.7–19.8)	<.001
in females (*n* = 1235)	20.8 (19.7–22.0)	18.3 (17.4–19.4)	17.4 (16.6–18.1)	16.6 (15.8–17.3)	<.001
Blood tests					
Haemoglobin (g/dl)	10.6 (9.9–11.3)	10.6 (9.8–11.3)	10.4 (9.7–11.1)	10.1 (9.4–10.8)	<.001
Serum albumin (g/dl)	3.9 (3.7–4.2)	3.8 (3.5–4.0)	3.7 (3.4–3.9)	3.5 (3.2–3.8)	<.001
Serum total cholesterol (mg/dl)	152 (132–178)	151 (129–180)	150 (130–176)	151 (126–165)	.189
Serum urea nitrogen (mg/dl)	70 (61–79)	64 (54–74)	61 (50–70)	60 (49–68)	<.001
Serum creatinine (mg/dl)	11.6 (10.3–13.1)	9.1 (7.6–10.8)	8.0 (7.0–9.2)	7.2 (5.7–8.3)	<.001
Serum CRP (mg/dl)	0.1 (0.0–0.2)	0.1 (0.1–0.3)	0.2 (0.1–0.5)	0.3 (0.1–1.1)	<.001
Corrected serum calcium (mg/dl)	9.5 (9.0–10.0)	9.3 (8.9–9.8)	9.3 (8.8–9.7)	9.4 (9.0–9.8)	.001
Serum phosphate (mg/dl)	5.1 (4.4–5.9)	4.7 (4.0–5.5)	4.6 (3.9–5.3)	4.3 (3.9–5.1)	<.001
Serum alkaline phosphatase (U/l)	221 (169–292)	239 (189–323)	260 (207–347)	269 (207–349)	<.001
Serum iPTH (pg/ml)	110 (51–231)	100 (43–195)	89 (44–186)	92 (52–150)	<.001
Medications					
Phosphate binders	1291 (89.7)	876 (79.5)	277 (64.3)	36 (62.1)	<.001
VDRAs	1085 (75.3)	753 (68.3)	276 (64.0)	34 (58.6)	<.001
Anti-hypertensives	891 (62.0)	718 (65.2)	288 (66.8)	38 (65.5)	.031

Values are presented as median (IQR) unless stated otherwise.

The Jonckheere–Terpstra test and the Cochran–Armitage test were used to calculate *P*-values for the trend of continuous variables and categorical variables, respectively. A two-tailed *P*-value <.05 was considered statistically significant.

Conversion factors for units: haemoglobin in g/dl to g/l, × 10; albumin in g/dl to g/l, × 10; total cholesterol in mg/dl to mmol/l, × 0.0259; urea nitrogen in mg/dl to mmol/l, × 0.357; creatinine in mg/dl to μmol/l, × 88.4; CRP in mg/dl to nmol/l, × 9.524; albumin-corrected calcium in mg/dl to mmol/l, × 0.25; phosphate in mg/dl to mmol/l, × 0.323; PTH hormone in pg/ml to ng/l, × 1.

### The risks for all-cause death, MACE and bone fracture in each group

**Figure 3:**
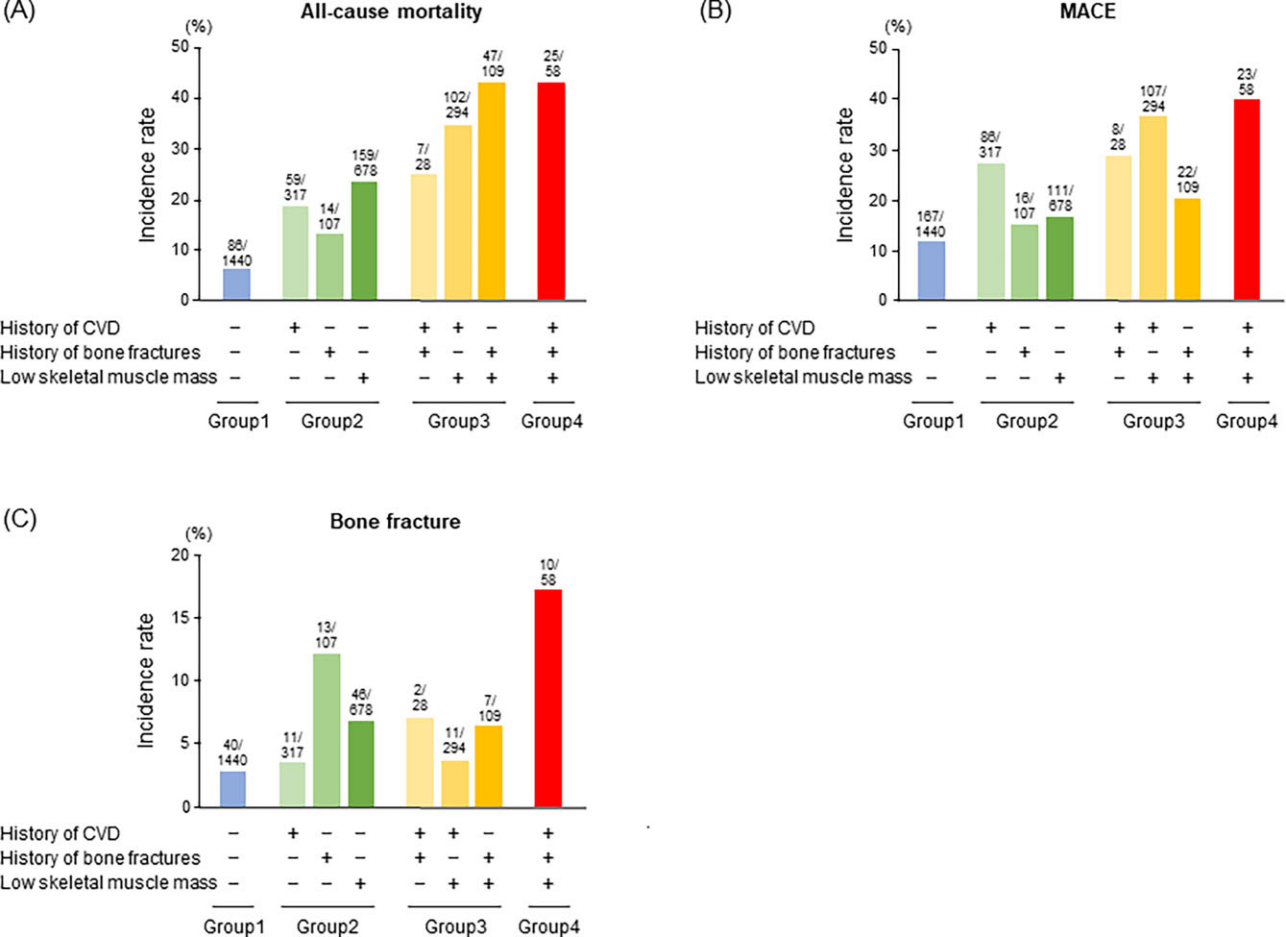
Incidence rates of **(A)** all-cause death, **(B)** MACE and **(C)** bone fracture in each group stratified by the combination of comorbidities. Patients were divided into four major groups according to the total number of the following complications: history of cardiovascular disease, history of bone fractures and the presence of low skeletal muscle mass.

**Figure 4:**
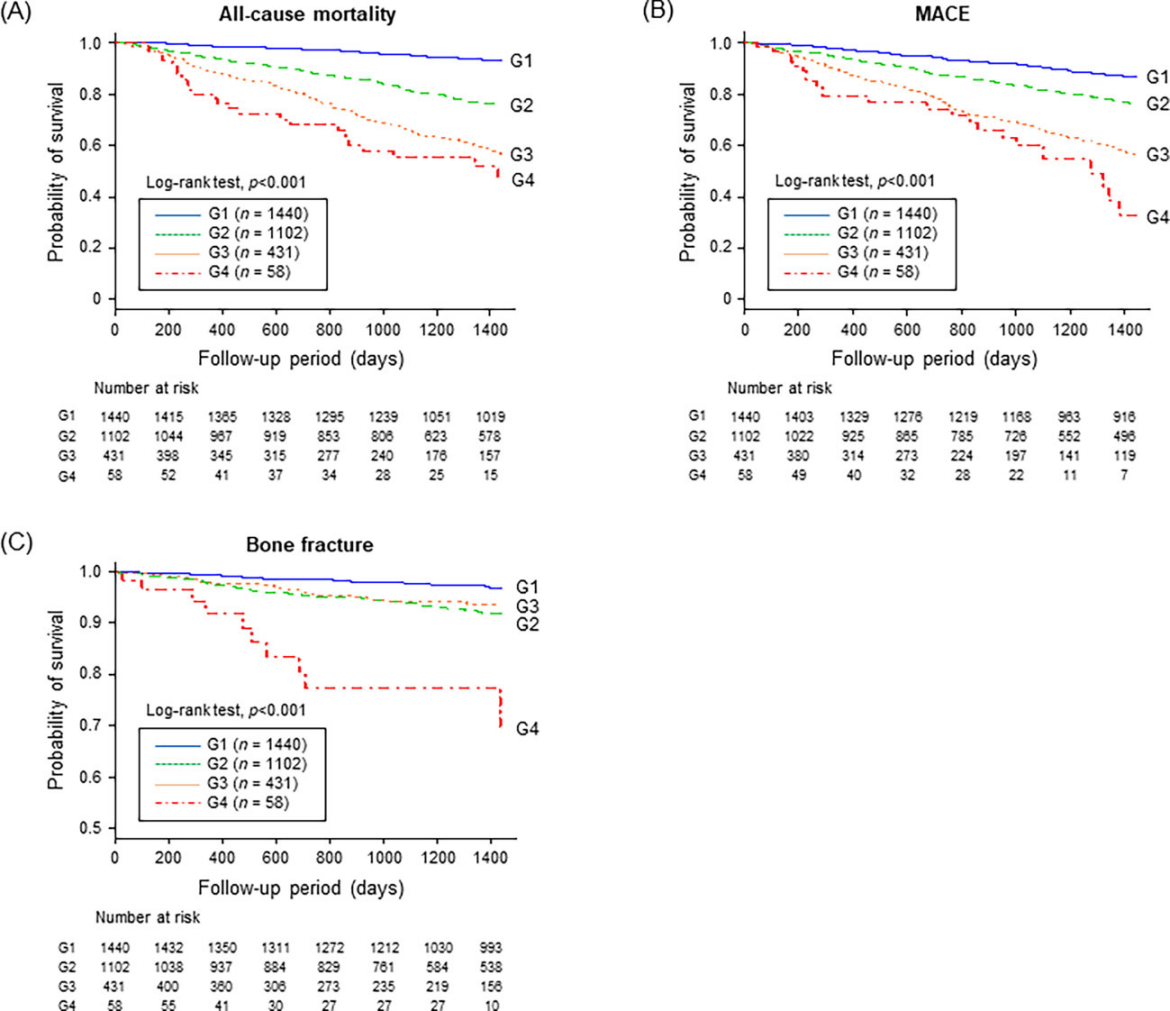
Kaplan–Meier curves for the incidence of **(A)** all-cause death, **(B)** MACE and **(C)** bone fracture in each group. Patients were divided into four groups according to the total number of the following complications: history of cardiovascular disease, history of bone fractures and the presence of low skeletal muscle mass. A logrank test was used to determine statistical differences. A two-tailed *P*-value <.05 was considered statistically significant. G: group.

During a median 4-year observation period, 499 all-cause deaths, 540 MACE and 140 bone fractures occurred. The incidence rates of all-cause death, MACE and bone fracture were the highest in patients in group 4 (Table 3). More detailed information on the incidence of outcomes during the observation period for each disease combination is shown in Fig. 3. Although the prevalence of a history of bone fracture was relatively low compared with other comorbidities, incidence rates of outcomes increased with the addition of a history of bone fracture (Fig. 3). Kaplan–Meier curves showed significantly higher incidence rates of each outcome in patients in groups 2, 3 and 4 compared with those in group 1 (all-cause death, *P* < .001; MACE, *P* < .001; bone fracture, *P* < .001; logrank test; Fig. 4).

**Table 3:** Relative risks for outcomes in each group (*N* = 3031).

			Age- and sex-adjusted model			Multivariable-adjusted model			Competing risk model		
Outcomes	*n*/*N* (%)	*P* for trend	HR (95% CI)	*P*-value	*P* for trend	HR (95% CI)	*P*-value	*P* for trend	HR (95% CI)	*P*-value	*P* for trend
All-cause death		<.001			<.001			<.001			
Group 1	86/1440 (6.0)		1.00 (reference)	–		1.00 (reference)	–				
Group 2	232/1102 (21.1)		2.59 (1.99–3.37)	<.001		2.16 (1.65–2.84)	<.001				
Group 3	156/431 (36.2)		4.20 (3.14–5.62)	<.001		3.10 (2.27–4.23)	<.001				
Group 4	25/58 (43.1)		5.11 (3.18–8.21)	<.001		3.11 (1.89–5.14)	<.001				
MACE		<.001			<.001			<.001			<.001
Group 1	167/1440 (11.6)		1.00 (reference)	–		1.00 (reference)	–		1.00 (reference)	–	
Group 2	213/1102 (19.3)		1.58 (1.27–1.96)	<.001		1.43 (1.15–1.79)	.002		1.40 (1.12–1.75)	.004	
Group 3	137/431 (31.8)		2.94 (2.28–3.80)	<.001		2.42 (1.85–3.18)	<.001		2.42 (1.70–2.94)	<.001	
Group 4	23/58 (39.7)		4.29 (2.70–6.81)	<.001		3.02 (1.88–4.87)	<.001		2.65 (1.67–4.22)	<.001	
Bone fracture		<.001			<.001			.002			.014
Group 1	40/1440 (2.8)		1.00 (reference)	–		1.00 (reference)	–		1.00 (reference)	–	
Group 2	70/1102 (6.4)		2.17 (1.42–3.32)	<.001		2.08 (1.36–3.20)	<.001		1.99 (1.29–3.08)	.002	
Group 3	20/431 (4.6)		1.62 (0.90–2.91)	.110		1.47 (0.80–2.68)	.213		1.32 (0.72–2.43)	.370	
Group 4	10/58 (17.2)		6.67 (3.11–14.27)	<.001		5.97 (2.72–13.10)	<.001		4.56 (1.99–10.49)	<.001	

Age- and sex-adjusted and multivariable-adjusted HRs for each outcome were calculated by the Cox proportional hazards risk model and the Fine–Gray proportional subdistribution hazards model with all-cause death as a competing risk (the competing risk model). The covariates for all-cause death and the development of MACE included age; sex; presence of diabetic nephropathy; dialysis vintage; dialysis time per session; systolic blood pressure; cardiothoracic ratio; nPCR; Kt/V for urea; BMI, blood haemoglobin; serum albumin, total cholesterol, corrected calcium, phosphate and alkaline phosphatase; log serum CRP, log serum iPTH and use of anti-hypertensives, phosphate binders and VDRAs. The covariates for the development of bone fracture included age; sex; presence of diabetic nephropathy; dialysis vintage; BMI; serum albumin, corrected calcium, phosphate and alkaline phosphatase; log serum CRP; log serum iPTH and use of phosphate binders and VDRAs. A two-tailed *P*-value <.05 was considered statistically significant.

In the age- and sex-adjusted and multivariable-adjusted Cox proportional hazards models, patients in groups 2, 3 and 4 showed significantly higher adjusted HRs for the incidence rates of all-cause death and MACE compared with those in group 1 as the reference (Table 3). In the competing risk model, patients in groups 2, 3 and 4 still showed significantly higher HRs for the development of MACE compared with those in group 1 (Table 3). In the Cox proportional hazards models and competing risk model, patients in groups 2 and 4 had significantly higher adjusted HRs for the incidence rates of bone fracture compared with those in group 1. Although patients in group 3 did not show significantly higher adjusted HRs for the incidence rates of bone fracture than those in group 1, trend analyses demonstrated the risk of bone fracture increased as the number of diseased organs increased.

### Subgroup analysis

**Figure 5:**
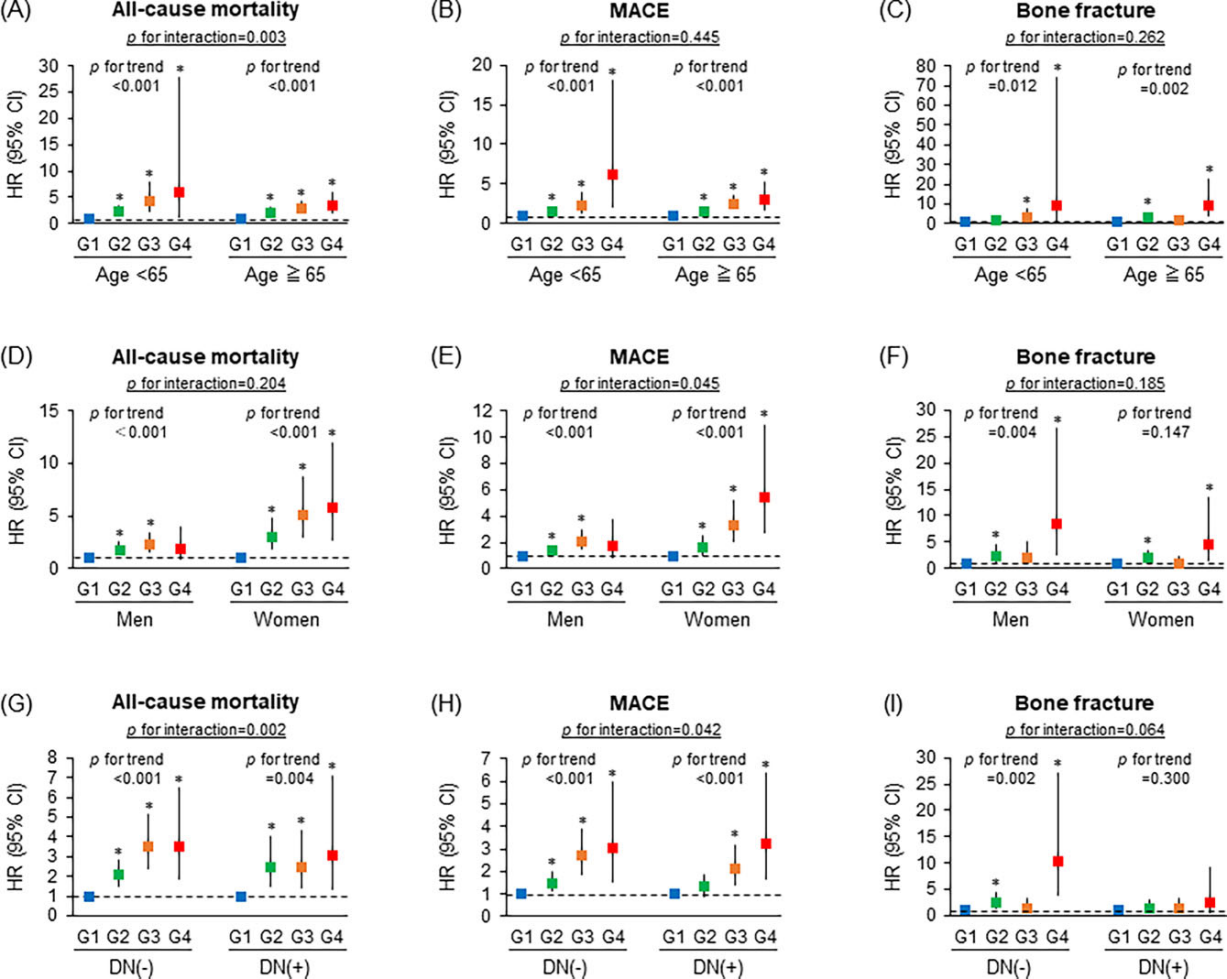
Relative risks for each outcome in the subgroup analyses. Multivariable-adjusted HRs and 95% CIs for all-cause death, MACE and bone fracture in the subgroups stratified by the baseline characteristics including age, sex and the presence of diabetic nephropathy. Filled squares denote point estimates of the HRs and error bars represent the 95% CIs. Patients were divided into four groups according to the total number of the following complications: history of cardiovascular disease, history of bone fractures and the presence of low skeletal muscle mass. The covariates for all-cause death and the development of MACE included age; sex; presence of DN; dialysis vintage; dialysis time per session; systolic blood pressure; cardiothoracic ratio; nPCR; Kt/V for urea; BMI; blood haemoglobin; serum albumin, total cholesterol, corrected calcium, phosphate and alkaline phosphatase; log serum CRP, log iPTH and use of antihypertensives, phosphate binders and VDRAs. The covariates for the development of bone fracture included age; sex; presence of DN; dialysis vintage; BMI; serum of albumin, corrected calcium, phosphate, and alkaline phosphatase; log serum CRP, log iPTH and the use of phosphate binders and VDRAs. The asterisk represents statistical significance when compared with the HR in group 1 as the reference. A two-tailed *P*-value <.05 was considered statistically significant. DN: diabetic nephropathy; G: group.

Next, subgroup analyses were performed to assess whether there was heterogeneity regarding the association between multiple organ disorders and baseline parameters regarding each outcome. The association between multiple organ disorders and all-cause mortality was strengthened in non-elderly patients compared with elderly patients, although the associations between multiple organ disorders and MACE and bone fracture were not affected by the patient’s age (Fig. 5A–C). The association between multiple organ disorders and MACE was strengthened in women compared with men, although associations between multiple organ disorders and all-cause mortality and bone fracture were not different across both sexes (Fig. 5D–F). The associations between combined organ disorders and all-cause mortality and MACE were strengthened in patients without diabetic nephropathy compared with patients with diabetic nephropathy, although the association between combined organ disorders and the development of bone fracture was not different regardless of the presence of diabetic nephropathy (Fig. 5G–I). Because malnutrition and inflammation are strong risk factors for adverse events in HD patients [27], termed malnutrition–inflammation complex syndrome (MICS) [28], we evaluated whether there was an interaction between multiple organ disorders and MICS markers regarding each outcome. The association between multiple organ disorders and MACE was accentuated in patients with inflammation compared with those without inflammation (Supplementary Figure S2).

## DISCUSSION

The present prospective cohort study of Japanese patients on maintenance HD demonstrated that patients with a history of cardiovascular disease and bone fractures or the presence of low skeletal muscle mass had a significantly higher prevalence of the other two morbidities. Furthermore, the greater the number of diseased organs the patients had, the higher the risks for all-cause mortality and the development of MACE and bone fracture, even after multivariable adjustment. Our study proposes the concept of the cardiovascular–bone–skeletal muscle axis, an extended concept of the bone-vascular axis should cover the musculoskeletal system, possessing multi-directional interplay between organs, which suggests that disturbances in these vital organs may place patients undergoing HD at an elevated risk of morbidity and mortality.

The cardiovascular system, bone and skeletal muscle have essential and independent roles in the body; however, the direct interplay between these organs to maintain their physiological structures and functions is not fully understood. Our data clearly showed that diseases in the three organs are closely linked (Fig. 2) and that patients on maintenance HD with multiple organ disorders had a greatly elevated risk of morbidity and mortality (Table 3). Such associations between these diseased organs indicate a potential multidirectional axis, termed the cardiovascular–bone–skeletal muscle axis, which may be more easily disrupted in patients on maintenance HD than in those in the general population. Disorders of the cardiovascular system, bone and skeletal muscle cause frailty, including locomotive dysfunction, with patients becoming bedridden, which is a burden on the healthcare system and individuals, especially in an elderly society [29, 30]. Therefore, maintaining this vital axis in patients on maintenance HD is important for preserving the activity of daily living or quality of life, achieving a better prognosis and reducing the burden on the healthcare system.

Direct and indirect interplay in the cardiovascular–bone–skeletal muscle axis should be discussed with patients. First, a history of cardiovascular disease, represented by ischaemic heart disease and stroke, reduces exercise tolerance or decreases physical capacity, leading to a decline in muscle strength and mass, i.e. sarcopenia. Furthermore, stroke or chronic brain ischaemia caused by atherosclerotic disease impairs cognitive and motor functions, which places patients at a high risk of falling, resulting in an increased risk for bone fractures [31]. Some studies have reported the direct impact of cardiovascular disorders on bone health. Collins *et al*. [32] described that peripheral artery disease was significantly associated with higher rates of hip bone loss and an increased risk for non-spine fractures in older men. Ischaemic osteoporosis may partly contribute to this interesting finding [4, 32–37].

Osteoporosis and renal osteodystrophy are critical in the pathophysiology of bone fractures in patients on maintenance HD [38]. Because bones have an essential role in maintaining the framework of the body, it is natural that bone fractures decrease patient activity, leading to sarcopenia. Furthermore, bones also have an indispensable role as a reservoir of minerals, including calcium and phosphate, and disturbances of mineral storage by decreased bone mineral density or inadequate bone turnover may cause ectopic calcification, including vascular calcification, an established risk factor for cardiovascular disease and mortality in the HD population [39–41].

According to Frost's mechanostat theory [3], a substantial degree of the mechanical load is required for normal bone modelling and remodelling, and a greater strain is needed to drive bone modelling, a process vital for bone growth. Consistent with this theory, Schipilow *et al.* [42] demonstrated that mechanical load in sports activity and muscle strength are significant predictors of bone quality in women. Conversely, Verschueren *et al.* [43] showed a significant association between reduced muscle mass and low bone mineral density in adult men, indicating an unfavourable interplay between sarcopenia and osteoporosis. These previous findings and our data confirm the significance of skeletal muscle quality and quantity for bone health and the vicious cycle in patients with sarcopenia and bone diseases such as osteoporosis and renal osteodystrophy.

The proposed cardiovascular–bone–skeletal muscle axis may partly involve humoral factors, which when secreted from each organ are candidates connecting interorgan health. For example, myokines such as irisin, secreted from skeletal muscle [2], have a protective role in the cardiovascular system and bone [44–47]. Furthermore, follistatin-like 1, produced by skeletal and cardiac muscle cells, and potentially regarded as a myokine and cardiokine [48], possesses cardioprotective effects [49, 50]. Such humoral mediators may have a fundamental role in maintaining interdependent organ health; however, once this vital interplay is disrupted, diseased organs affect each other adversely, generating a vicious cycle in this vital axis and worsening mortality in HD patients.

Patients with cardiovascular diseases, bone fractures and sarcopenia are more likely to develop MICS, which in turn mediates these organ disorders, increasing the risk of all-cause and cardiovascular mortality [51]. The increased production of inflammatory cytokines and oxidative stress are shared pathomechanisms responsible for concomitant disorders in the cardiovascular, bone and skeletal muscle systems. It is reasonable to speculate that MICS is an effect modifier regarding the association between multiple organ disorders and mortality or cardiovascular disease events. In the subgroup analysis, associations between multiple organ disorders and MACE were strengthened in patients with higher serum CRP (Supplementary Figure S2E). The results confirm the potential involvement of malnutrition and chronic inflammation in the pathogenesis of disorders in the cardiovascular–bone–skeletal muscle axis and encourage medical practitioners to manage malnutrition and chronic inflammation appropriately in HD patients.

In the subgroup analyses, associations between combined organ disorders and all-cause mortality were strengthened in non-elderly patients and those without diabetic nephropathy (Fig. 5A and G). Furthermore, associations between combined organ disorders and MACE were strengthened in women and patients without diabetic nephropathy (Fig. 5E and H). Although the precise biological mechanisms involved should be clarified in future studies, our data suggest that the management of disturbances in the cardiovascular–bone–skeletal muscle axis should be strengthened in patients with unmodifiable background factors including non-elderly, female sex and non-diabetic status.

This study had several limitations. First, we measured the modified creatinine index, which was used for the definition of low skeletal muscle mass, only once at baseline, therefore there is a possibility of misclassification bias. The skeletal muscle volume might have changed during the observation period, depending on the patient's nutritional status and exercise. Second, the definition of low skeletal muscle in our study was based on a previous study examining the discriminative ability of the modified creatinine index to diagnose sarcopenia [24]. Additionally, muscle strength or physical performance, which are critical for a diagnosis of sarcopenia, were not considered in the present study. The impact of low skeletal muscle mass on outcomes may vary depending on the different cut-off values of the modified creatinine index, criteria or diagnostic modalities. Third, we did not distinguish the cause and site of bone fracture in this study. Factors derived from the diseased cardiovascular system, bone and skeletal muscle may have an inconsistent effect on different bone sites. Fourth, the prevalence of a history of bone fracture was relatively small compared with other comorbidities. The role of bone disorders in the cardiovascular–bone–skeletal muscle axis may not have been accurately assessed in this study, partly due to the underdiagnosis of bone disorders: patients with asymptomatic bone fractures such as lumbar vertebral fractures or low bone mineral density without bone fractures were not included in the present study. Finally, although we attempted to rigorously adjust for potential confounders from the baseline characteristics, known and unknown residual confounders may have affected the results. Despite these limitations, we believe that our study provides valuable information to aid in understanding the importance of the cardiovascular–bone–skeletal muscle axis in healthy and unhealthy HD patients.

In conclusion, our data suggest that accumulated disturbances in cardiovascular, bone and skeletal muscle health are significantly associated with higher risks for all-cause mortality and the development of MACE and bone fracture in patients on maintenance HD. These results shed new light on the concept of the cardiovascular–bone–skeletal muscle axis in the HD population. Because cardiovascular risk factors, bone status and skeletal muscle mass are modifiable targets, future studies are needed to elucidate whether pharmacological or non-pharmacological treatments targeting these organs improve outcomes in the HD population.

## Supplementary Material

sfae154_Supplemental_File

## Data Availability

The data underlying this article will be shared upon reasonable request to the corresponding author.

## References

[1] Kirk B, Feehan J, Lombardi G et al. Muscle, bone, and fat crosstalk: the biological role of myokines, osteokines, and adipokines. Curr Osteoporos Rep 2020;18:388–400. 10.1007/s11914-020-00599-y 32529456

[2] Severinsen MCK, Pedersen BK. Muscle–organ crosstalk: the emerging roles of myokines. Endocr Rev 2020;41:594–609. 10.1210/endrev/bnaa016 32393961 PMC7288608

[3] Frost HM . The mechanostat: a proposed pathogenic mechanism of osteoporoses and the bone mass effects of mechanical and nonmechanical agents. Bone Miner 1987;2:73–85. 3333019

[4] Reeve J, Arlot M, Wootton R et al. Skeletal blood flow, iliac histomorphometry, and strontium kinetics in osteoporosis: a relationship between blood flow and corrected apposition rate. J Clin Endocrinol Metab 1988;66:1124–31. 10.1210/jcem-66-6-1124 3372678

[5] Sivaraj KK, Adams RH. Blood vessel formation and function in bone. Development 2016;143:2706–15. 10.1242/dev.136861 27486231

[6] Mace ML, Egstrand S, Morevati M et al. New insights to the crosstalk between vascular and bone tissue in chronic kidney disease-mineral and bone disorder. Metabolites 2021;11:849. 10.3390/metabo11120849 34940607 PMC8708186

[7] Nitta K, Goto S, Masakane I et al. Annual dialysis data report for 2018, JSDT Renal Data Registry: survey methods, facility data, incidence, prevalence, and mortality. Ren Replace Ther 2020;6:41. 10.1186/s41100-020-00286-9

[8] Cozzolino M, Mangano M, Stucchi A et al. Cardiovascular disease in dialysis patients. Nephrol Dial Transplant 2018;33:28–34. 10.1093/ndt/gfy174 PMC6168816 30281132

[9] Iseri K, Carrero JJ, Evans M et al. Major fractures after initiation of dialysis: incidence, predictors and association with mortality. Bone 2020;133:115242. 10.1016/j.bone.2020.115242 31958531

[10] Isoyama N, Qureshi AR, Avesani CM et al. Comparative associations of muscle mass and muscle strength with mortality in dialysis patients. Clin J Am Soc Nephrol 2014;9:1720–8. 10.2215/CJN.10261013 25074839 PMC4186520

[11] Johansen KL, Delgado C, Kaysen GA et al. Frailty among patients receiving haemodialysis: evolution of components and associations with mortality. J Gerontol A Biol Sci Med Sci 2019;74:380–6. 10.1093/gerona/gly206 30192916 PMC6376100

[12] Haruyama N, Nakayama M, Yamada S et al. History of fragility fracture is associated with cardiovascular mortality in haemodialysis patients: the Q-Cohort Study. J Bone Miner Metab 2024;42:253–63. 38509305 10.1007/s00774-024-01501-x

[13] Veronese N, Stubbs B, Crepaldi G et al. Relationship between low bone mineral density and fractures with incident cardiovascular disease: a systematic review and meta-analysis. J Bone Miner Res 2017;32:1126–35. 10.1002/jbmr.3089 28138982 PMC5417361

[14] Thompson B, Towler DA. Arterial calcification and bone physiology: role of the bone-vascular axis. Nat Rev Endocrinol 2012;8:529–43. 10.1038/nrendo.2012.36 22473330 PMC3423589

[15] Evenepoel P, Opdebeeck B, David K et al. Bone-vascular axis in chronic kidney disease. Adv Chronic Kidney Dis 2019;26:472–83. 10.1053/j.ackd.2019.09.006 31831125

[16] Gu W, Wang Z, Sun Z et al. Role of NFATc1 in the bone-vascular axis calcification paradox. J Cardiovasc Pharmacol 2020;75:200–7. 10.1097/FJC.0000000000000788 31868826

[17] Arase H, Yamada S, Yotsueda R et al. Modified creatinine index and risk for cardiovascular events and all-cause mortality in patients undergoing haemodialysis: the Q-Cohort study. Atherosclerosis 2018;275:115–23. 10.1016/j.atherosclerosis.2018.06.001 29890446

[18] Yamada S, Taniguchi M, Tokumoto M et al. Modified creatinine index and the risk of bone fracture in patients undergoing haemodialysis: the Q-Cohort study. Am J Kidney Dis 2017;70:270–80. 10.1053/j.ajkd.2017.01.052 28450093

[19] Eriguchi R, Taniguchi M, Ninomiya T et al. Hyporesponsiveness to erythropoiesis-stimulating agent as a prognostic factor in Japanese haemodialysis patients: the Q-Cohort study. J Nephrol 2015;28:217–25. 10.1007/s40620-014-0121-9 25080399

[20] Yamada S, Tsuruya K, Taniguchi M et al. Association between serum phosphate levels and stroke risk in patients undergoing haemodialysis: the Q-Cohort study. Stroke 2016;47:2189–96. 10.1161/STROKEAHA.116.013195 27507862

[21] Arase H, Yamada S, Hiyamuta H et al. Modified creatinine index and risk for long-term infection-related mortality in haemodialysis patients: ten-year outcomes of the Q-Cohort Study. Sci Rep 2020;10:1241. 10.1038/s41598-020-58181-6 31988325 PMC6985259

[22] Tanaka S, Ninomiya T, Taniguchi M et al. Impact of blood urea nitrogen to creatinine ratio on mortality and morbidity in haemodialysis patients: the Q-Cohort study. Sci Rep 2017;7:14901. 10.1038/s41598-017-14205-2 29097750 PMC5668292

[23] Canaud B, Granger Vallee A, Molinari N et al. Creatinine index as a surrogate of lean body mass derived from urea kt/V, pre-dialysis serum levels and anthropometric characteristics of haemodialysis patients. PLoS One 2014;9:e93286. 10.1371/journal.pone.0093286 24671212 PMC3966881

[24] Kakita D, Matsuzawa R, Yamamoto S et al. Simplified discriminant parameters for sarcopenia among patients undergoing haemodialysis. J Cachexia Sarcopenia Muscle 2022;13:2898–907. 10.1002/jcsm.13078 36058558 PMC9745501

[25] Chen LK, Woo J, Assantachai P et al. Asian Working Group for Sarcopenia: 2019 consensus update on sarcopenia diagnosis and treatment. J Am Med Dir Assoc 2020;21:300–7.e2. 10.1016/j.jamda.2019.12.012 32033882

[26] Kittiskulnam P, Chertow GM, Carrero JJ et al. Sarcopenia and its individual criteria are associated, in part, with mortality among patients on haemodialysis. Kidney Int 2017;92:238–47. 10.1016/j.kint.2017.01.024 28318630 PMC5483392

[27] Yamada S, Arase H, Yoshida H et al. Malnutrition-inflammation complex syndrome and bone fractures and cardiovascular disease events in patients undergoing haemodialysis: the Q-cohort study. Kidney Med 2022;4:100408. 10.1016/j.xkme.2022.100408 35386605 PMC8978069

[28] Kalantar-Zadeh K, Ikizler TA, Block G et al. Malnutrition-inflammation complex syndrome in dialysis patients: causes and consequences. Am J Kidney Dis 2003;42:864–81. 10.1016/j.ajkd.2003.07.016 14582032

[29] Johansen KL, Dalrymple LS, Glidden D et al. Association of performance-based and self-reported function-based definitions of frailty with mortality among patients receiving haemodialysis. Clin J Am Soc Nephrol 2016;11:626–32. 10.2215/CJN.03710415 26792529 PMC4822658

[30] Kutner NG, Zhang R, Huang Y et al. Gait speed and mortality, hospitalization, and functional status change among haemodialysis patients: a US Renal Data System Special study. Am J Kidney Dis 2015;66:297–304. 10.1053/j.ajkd.2015.01.024 25824124 PMC4516659

[31] Harvey L, Mitchell R, Brodaty H et al. Differing trends in fall-related fracture and non-fracture injuries in older people with and without dementia. Arch Gerontol Geriatr 2016;67:61–7. 10.1016/j.archger.2016.06.014 27434743

[32] Collins TC, Ewing SK, Diem SJ et al. Peripheral arterial disease is associated with higher rates of hip bone loss and increased fracture risk in older men. Circulation 2009;119:2305–12. 10.1161/CIRCULATIONAHA.108.820993 19380619 PMC2766264

[33] Brenneise CV, Squier CA. Blood flow in maxilla and mandible of normal and atherosclerotic rhesus monkeys. J Oral Pathol 1985;14:800–8. 10.1111/j.1600-0714.1985.tb00470.x 3932619

[34] Yamada S, Nakano T, Kitamura H et al. A case of ischemic osteopathy in a haemodialysis patient with advanced peripheral artery disease. CEN Case Rep 2020;9:89–90. 10.1007/s13730-019-00425-0 31637589 PMC6990279

[35] London GM . Soft bone – hard arteries: a link? Kidney Blood Press Res 2011;34:203–8. 10.1159/000327004 21691122

[36] Alagiakrishnan K, Juby A, Hanley D et al. Role of vascular factors in osteoporosis. J Gerontol A Biol Sci Med Sci 2003;58:362–6. 10.1093/gerona/58.4.M362 12663699

[37] Griffith JF, Yeung DK, Tsang PH et al. Compromised bone marrow perfusion in osteoporosis. J Bone Miner Res 2008;23:1068–75. 10.1359/jbmr.080233 18302498

[38] Pimentel A, Ureña-Torres P, Zillikens MC et al. Fractures in patients with CKD-diagnosis, treatment, and prevention: a review by members of the European Calcified Tissue Society and the European Renal Association of Nephrology Dialysis and Transplantation. Kidney Int 2017;92:1343–55. 10.1016/j.kint.2017.07.021 28964571

[39] London GM, Guérin AP, Marchais SJ et al. Arterial media calcification in end-stage renal disease: impact on all-cause and cardiovascular mortality. Nephrol Dial Transplant 2003;18:1731–40. 10.1093/ndt/gfg414 12937218

[40] Blacher J, Guerin AP, Pannier B et al. Arterial calcifications, arterial stiffness, and cardiovascular risk in end-stage renal disease. Hypertension 2001;38:938–42. 10.1161/hy1001.096358 11641313

[41] Adragao T, Pires A, Lucas C et al. A simple vascular calcification score predicts cardiovascular risk in haemodialysis patients. Nephrol Dial Transplant 2004;19:1480–8. 10.1093/ndt/gfh217 15034154

[42] Schipilow JD, Macdonald HM, Liphardt AM et al. Bone micro-architecture, estimated bone strength, and the muscle-bone interaction in elite athletes: an HR-pQCT study. Bone 2013;56:281–9. 10.1016/j.bone.2013.06.014 23800515

[43] Verschueren S, Gielen E, O'Neill TW et al. Sarcopenia and its relationship with bone mineral density in middle-aged and elderly European men. Osteoporos Int 2013;24:87–98. 10.1007/s00198-012-2057-z 22776861

[44] Shimba Y, Togawa H, Senoo N et al. Skeletal muscle-specific PGC-1α overexpression suppresses atherosclerosis in apolipoprotein E-knockout mice. Sci Rep 2019;9:4077. 10.1038/s41598-019-40643-1 30858489 PMC6411944

[45] Zhang Y, Song H, Zhang Y et al. Irisin inhibits atherosclerosis by promoting endothelial proliferation through microRNA126-5p. J Am Heart Assoc 2016;5:e004031. 10.1161/JAHA.116.004031 27671318 PMC5079047

[46] Colaianni G, Sanesi L, Storlino G et al. Irisin and bone: from preclinical studies to the evaluation of its circulating levels in different populations of human subjects. Cells 2019;8:451. 10.3390/cells8050451 31091695 PMC6562988

[47] Qiao X, Nie Y, Ma Y et al. Irisin promotes osteoblast proliferation and differentiation via activating the MAP kinase signaling pathways. Sci Rep 2016;6:18732. 10.1038/srep18732 26738434 PMC4704023

[48] Shimano M, Ouchi N, Walsh K. Cardiokines: recent progress in elucidating the cardiac secretome. Circulation 2012;126:e327–32. 10.1161/CIRCULATIONAHA.112.150656 23169257

[49] Ouchi N, Oshima Y, Ohashi K et al. Follistatin-like 1, a secreted muscle protein, promotes endothelial cell function and revascularization in ischemic tissue through a nitric-oxide synthase-dependent mechanism. J Biol Chem 2008;283:32802–11. 10.1074/jbc.M803440200 18718903 PMC2583310

[50] Oshima Y, Ouchi N, Sato K et al. Follistatin-like 1 is an Akt-regulated cardioprotective factor that is secreted by the heart. Circulation 2008;117:3099–108. 10.1161/CIRCULATIONAHA.108.767673 18519848 PMC2679251

[51] Yamada S, Tsuruya K, Kitazono T et al. Emerging cross-talks between chronic kidney disease-mineral and bone disorder (CKD-MBD) and malnutrition-inflammation complex syndrome (MICS) in patients receiving dialysis. Clin Exp Nephrol 2022;26:613–29. 10.1007/s10157-022-02216-x 35353283 PMC9203392

