# Supplementary material for: Disturbance in the potential cardiovascular–bone–skeletal muscle axis and morbidity and mortality in patients undergoing haemodialysis: the Q-Cohort Study
Source: Clin Kidney J. 2024 Jun 17;17(6):sfae154. doi: 10.1093/ckj/sfae154 (PMC11196899; doi:10.1093/ckj/sfae154)
Supplement: sfae154_Supplemental_File [file sfae154_supplemental_file.pdf]

SUPPLEMENTARY MATERIAL

Disturbance in the potential cardiovascular-bone-skeletal muscle axis and morbidity and mortality in patients undergoing haemodialysis: the Q-Cohort Study

Hokuto Arase M.D.[a, b], Shunsuke Yamada M.D., Ph.D.[a], Masatomo Taniguchi M.D., Ph.D.[c], Hiroaki Ooboshi M.D., Ph.D.[d], Kazuhiko Tsuruya M.D., Ph.D.[e], Takanari Kitazono M.D., Ph.D.[a], and Toshiaki Nakano M.D., Ph.D.[a]

a Department of Medicine and Clinical Science, Graduate School of Medical Sciences, Kyushu University, 3-1-1 Maidashi, Higashi-Ku, Fukuoka 8128582, Japan

b Department of Nephrology, NHO Fukuokahigashi Medical Center, 1-1-1 Chidori, Koga 8113195, Japan

c Fukuoka Renal Clinic, 4-6-20 Watanabe-dori, Chuo-Ku, Fukuoka 810-0004, Japan

d Department of Internal Medicine, Fukuoka Dental College, 2-15-1 Tamura, Sawara-Ku, Fukuoka 8140193, Japan

e Department of Nephrology, Nara Medical University, 840 Shijo-Cho, Kashihara, Nara 6348521, Japan

***Corresponding author**

Shunsuke Yamada, M.D., Ph.D.

Department of Medicine and Clinical Science, Graduate School of Medical Sciences,

Kyushu University, 3-1-1 Maidashi, Higashi-Ku, Fukuoka 8128582, Japan

Tel.: +81-92-642-5843; Fax: +81-92-642-5846

E-mail: ana65641@nifty.com

Supplementary Manuscript

Materials and Methods

Routine parameters, including blood haemoglobin level, serum level of albumin, total cholesterol, C-reactive protein, urea nitrogen, creatinine, calcium, phosphate, and alkaline phosphatase, were measured using an auto-analyzer with standard procedures at different laboratories. Serum parathyroid hormone (PTH) levels were measured as whole or intact PTH and serum levels of whole PTH were converted into those of intact PTH as follows: intact PTH (pg/mL) = 1.7 × whole PTH (pg/mL)[1]. Payne's formula was used to calculate corrected serum calcium levels when serum albumin levels were less than 4 g/dL: corrected serum calcium = serum calcium level + (4 − serum albumin level)[2].

Reference

1. Kazama JJ. Japanese Society of Dialysis Therapy treatment guidelines for secondary hyperparathyroidism. Ther Apher Dial 2007;11 Suppl 1:S44-7.

2. Payne RB, Little AJ, Williams RB, Milner JR. Interpretation of serum calcium in patients with abnormal serum proteins. Br Med J 1973;4(5893):643-6.

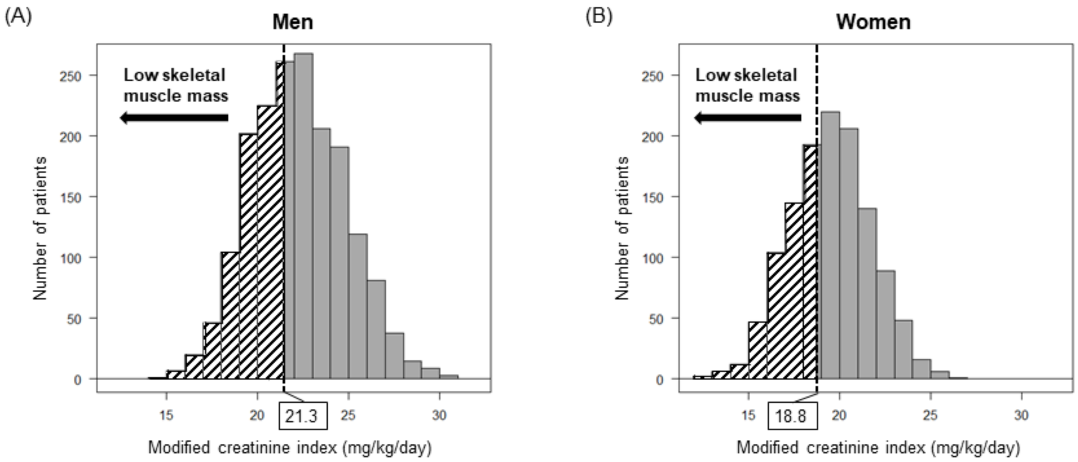

Supplementary Figure Legends

Supplementary Figure 1. Distribution of the modified creatinine index in (A) men and (B) women. Patients with low skeletal muscle mass were defined as those with a low modified creatinine index below the threshold at baseline. In this study, the cut-off values of the modified creatinine index were 21.3 mg/kg/day for men and 18.8 mg/kg/day for women, respectively.

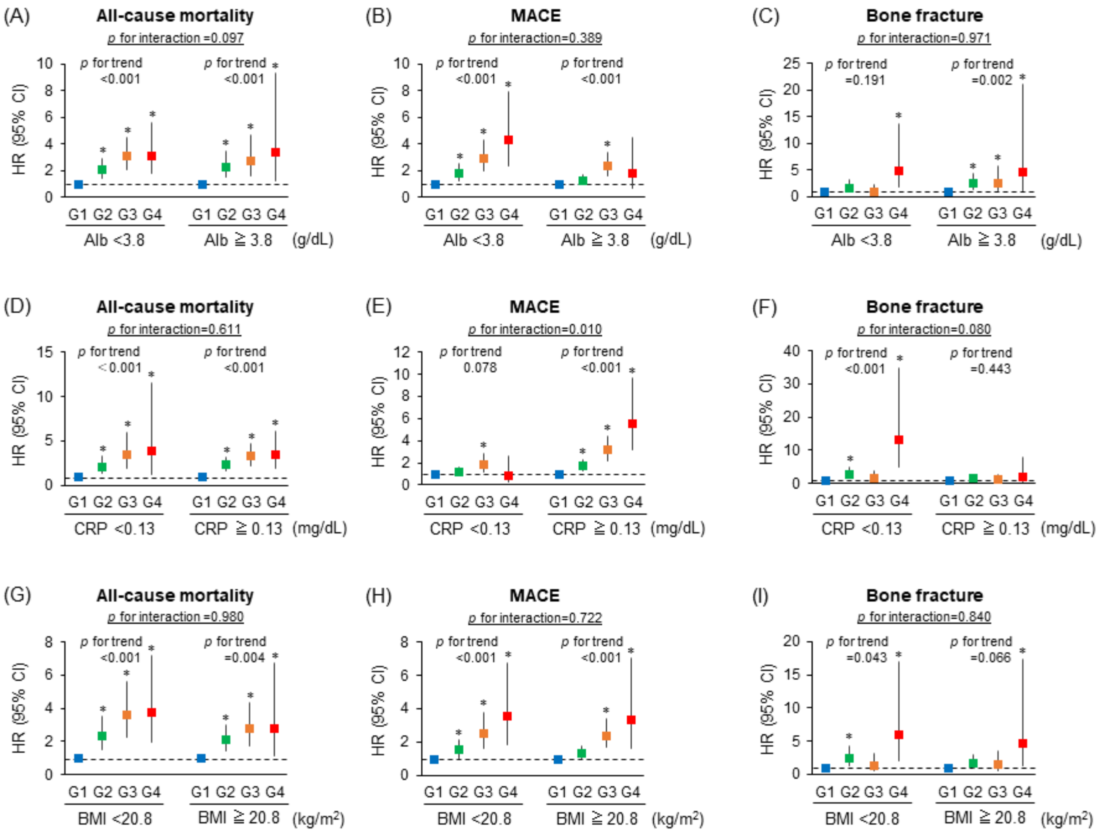

Supplementary Figure 2. Relative risks for each outcome in the subgroup analyses. Multivariable-adjusted hazard ratios (HR) and 95% confidence intervals (CI) for all-cause death, MACE, and bone fracture in the subgroups stratified by the baseline characteristics including serum levels of albumin and C-reactive protein, and body mass index. Filled squares denote point estimates of the HR, and error bars represent the 95% CI. Patients were divided into four groups according to the total number of the following three complications at baseline: histories of cardiovascular disease and bone fractures, and the presence of low skeletal muscle mass. The covariates for all-cause death and the development of MACE included age, sex, presence of DN, dialysis

vintage, dialysis time per session, systolic blood pressure, cardiothoracic ratio, nPCR, Kt/V for urea, body mass index, blood haemoglobin, serum level of albumin, total cholesterol, corrected calcium, phosphate, and alkaline phosphatase, log serum C-reactive protein level, log serum intact parathyroid hormone level, and use of anti-hypertensives, phosphate binders, and VDRAs. The covariates for the development of bone fracture included age, sex, presence of DN, dialysis vintage, body mass index, serum level of albumin, corrected calcium, phosphate, and alkaline phosphatase, log serum C-reactive protein level, log serum intact parathyroid hormone level, and use of phosphate binders and VDRAs. The asterisk represents statistical significance when compared with the HR in Group 1 as the reference. A two-tailed *p*-value less than 0.05 was considered statistically significant. Abbreviations: Alb, albumin; BMI, body mass index; CRP, C-reactive protein; DN, diabetic nephropathy; G, group; MACE, major adverse cardiovascular events; nPCR, normalized protein catabolic rate; VDRAs, vitamin D receptor activators.
